# Supplementary material for: The Small RNA Universe of Capitella teleta
Source: Front Mol Biosci. 2022 Feb 25;9:802814. doi: 10.3389/fmolb.2022.802814 (PMC8915122; doi:10.3389/fmolb.2022.802814)
Supplement: Supplementary file 1 [file DataSheet1.ZIP › Supplement/known/CAPTEscaffold_291_16145.pdf]

Provisional ID	:	CAPTEscaffold_291_16145
Score total	:	3505105.4
Score for star read(s)	:	3.9
Score for read counts	:	3505095.3
Score for mfe	:	1.6
Score for randfold	:	1.6
Score for cons. seed	:	3
Total read count	:	6875105
Mature read count	:	6874403
Loop read count	:	0
Star read count	:	702

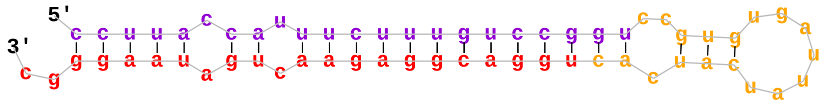

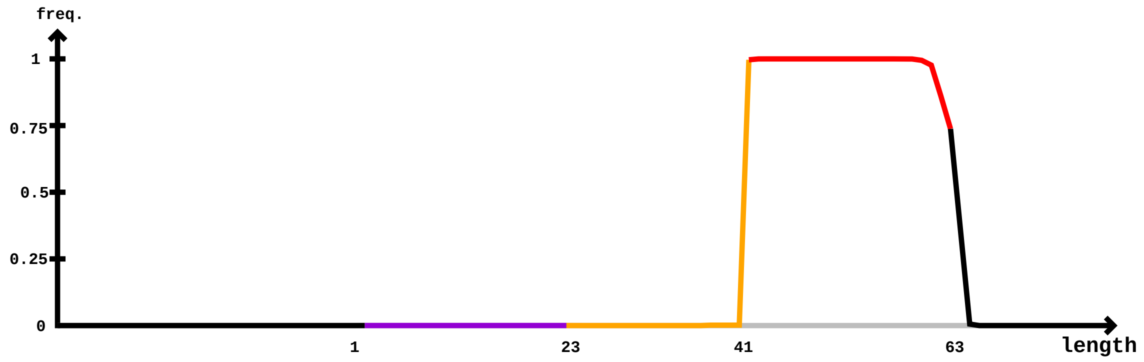

Star **Mature**

```
5'- gcguucgcugcaucacucgaucgcacgauuccuuaccauuucuuuguccgguccgugugauuaucaucacuggacggagaacugauaagggccugugacgaccacgag  -3'  obs
    gcguucgcugcaucacucgaucgcacgauuccuuaccauuucuuuguccgguccgugugauuaucaucacuggacggagaacugauaagggccugugacgaccacgag       exp
    gcguucgcugcaucacucgaucgcacgauuccuuaccauuucuuuguccgguccgugugauuaucaucacuggacggagaacugauaagggccugugacgaccacgag       known
    (((.....)))....((((.(((((((..((((((.((.(((((((((((((..(((.......))).))))))))))))).)).))))))..)))).)))...))))  reads  mm  sample
    ...........................auuccuuaccauuucuuugucc...........................................................  3      0   seq
    .............................uccuuaccauuucuuuguccgg.........................................................  1      0   seq
    ..............................ccuuaccauuucuuuguccgg.........................................................  12     0   seq
    ..............................ccAuaccauuucuuuguccggu........................................................  1      1   seq
    ..............................ccuuacAauuucuuuguccggu........................................................  3      1   seq
    ..............................ccuuaccauuucuuuguccggG........................................................  4      1   seq
    ..............................Ucuuaccauuucuuuguccggu........................................................  1      1   seq
    ..............................ccuuaccauuucuuuguccggu........................................................  536    0   seq
    ..............................ccuuaccauuuUuuuguccggu........................................................  1      1   seq
    ..............................ccuuaccauuucuAuguccggu........................................................  1      1   seq
    ..............................ccuuaccauuucuuuguccAgu........................................................  2      1   seq
    ..............................ccuuaccauuucuuuguccgguU.......................................................  4      1   seq
    ..............................ccuuaccauuucuuuguccgguc.......................................................  8      0   seq
    ..............................ccuuaccauuucuuuguccggucU......................................................  2      1   seq
    ...............................cuuaccauuucuuuguccgg.........................................................  19     0   seq
    ...............................cuuacAauuucuuuguccgg.........................................................  1      1   seq
    ...............................cuuaccauuucuuuguccggu........................................................  5      0   seq
    ...............................cuuaccauuucuuuguccgguc.......................................................  79     0   seq
    ...............................cuuaccauuucuuuguccggucU......................................................  14     1   seq
    ...............................cuuaccauuucuuuguccggucA......................................................  4      1   seq
    ................................uuaccauuucuuuguccggu........................................................  1      0   seq
    ................................uuaccauuucuuuguccgguc.......................................................  1      0   seq
    ................................uuaccauuucuuuguccggucU......................................................  1      1   seq
    .................................uaccauuucuuuguccgguccgug...................................................  1      0   seq
    .................................................................aucacuggacggagaacugau......................  2      0   seq
    ..................................................................ucacuNgacggagaacuga.......................  1      1   seq
    ..................................................................ucacuggacggagaacugG.......................  1      1   seq
    ..................................................................ucacAggacggagaacuga.......................  1      1   seq
    ..................................................................ucacuggacggagaacuga.......................  167    0   seq
    ..................................................................uUacuggacggagaacuga.......................  1      1   seq
    ..................................................................Acacuggacggagaacugau......................  9      1   seq
    ..................................................................ucacuggacggagaUcugau......................  1      1   seq
```

Star **Mature**

```
gcguucgcugcaucacucgaucgcacgauuccuuaccauuucuuuguccgguccgugugauuaucaucacuggacggagaacugauaagggccugugacgaccacgag
..................................................................Ncacuggacggagaacugau......................  1     1  seq
..................................................................ucacuggacggagaacCgau......................  1     1  seq
..................................................................ucacuAgacggagaacugau......................  5     1  seq
..................................................................ucacuggacAgagaacugau......................  1     1  seq
..................................................................ucacuggacggagaacAgau......................  1     1  seq
..................................................................ucacAggacggagaacugau......................  1     1  seq
..................................................................ucacuggacggGgaacugau......................  1     1  seq
..................................................................uAacuggacggagaacugau......................  1     1  seq
..................................................................ucacuggacggagaacugau......................  1146  0  seq
..................................................................uUacuggacggagaacugau......................  2     1  seq
..................................................................ucacuggaUggagaacugau......................  1     1  seq
..................................................................ucacuggacggagaacugaua.....................  1652  0  seq
..................................................................ucacuggacggagaacugaAa.....................  3     1  seq
..................................................................ucacuggacggagaaAugaua.....................  1     1  seq
..................................................................ucacAggacggagaacugaua.....................  3     1  seq
..................................................................ucacuggGcggagaacugaua.....................  1     1  seq
..................................................................ucacugAacggagaacugaua.....................  1     1  seq
..................................................................ucacuggaUggagaacugaua.....................  2     1  seq
..................................................................ucacuggacggagaacuAaua.....................  3     1  seq
..................................................................Acacuggacggagaacugaua.....................  10    1  seq
..................................................................ucacuggacUgagaacugaua.....................  1     1  seq
..................................................................Gcacuggacggagaacugaua.....................  1     1  seq
..................................................................ucacuggacggagaaUugaua.....................  1     1  seq
..................................................................uUacuggacggagaacugaua.....................  1     1  seq
..................................................................ucacuggacggagaacugUua.....................  2     1  seq
..................................................................ucacuggacggagaacuCaua.....................  1     1  seq
..................................................................ucacuggacCgagaacugaua.....................  1     1  seq
..................................................................ucacuggacggGgaacugaua.....................  1     1  seq
..................................................................ucacuggacAgagaacugaua.....................  3     1  seq
..................................................................ucacuggacggagaacugauU.....................  1     1  seq
..................................................................ucacuAgacggagaacugaua.....................  3     1  seq
..................................................................ucacuggacUgagaacugauaa....................  2     1  seq
..................................................................ucacuggacggagaacugaAaa....................  2     1  seq
..................................................................ucacuggacggagaacuAauaa....................  6     1  seq
..................................................................ucacAggacggagaacugauaa....................  13    1  seq
..................................................................ucacuggacggagaaUugauaa....................  1     1  seq
..................................................................ucacuggacgAagaacugauaa....................  1     1  seq
..................................................................ucacuggacggagaacuUauaa....................  1     1  seq
..................................................................ucacuggaUggagaacugauaa....................  3     1  seq
..................................................................ucacuggacggagaacAgauaa....................  1     1  seq
..................................................................ucacuggacggagaacugauaa....................  5435  0  seq
..................................................................ucacugAacggagaacugauaa....................  1     1  seq
..................................................................ucacuCgacggagaacugauaa....................  1     1  seq
..................................................................ucacuggacggagaacugGuaa....................  1     1  seq
..................................................................ucGcuggacggagaacugauaa....................  1     1  seq
..................................................................ucacGggacggagaacugauaa....................  2     1  seq
..................................................................ucacuggacgCagaacugauaa....................  1     1  seq
..................................................................Gcacuggacggagaacugauaa....................  3     1  seq
..................................................................ucacuggacggGgaacugauaa....................  2     1  seq
..................................................................ucacuggacggagaacugauaC....................  1     1  seq
..................................................................ucacuggaGggagaacugauaa....................  1     1  seq
..................................................................uUacuggacggagaacugauaa....................  10    1  seq
..................................................................ucacuggCcggagaacugauaa....................  1     1  seq
..................................................................ucacuAgacggagaacugauaa....................  14    1  seq
..................................................................Acacuggacggagaacugauaa....................  22    1  seq
..................................................................ucacuggacAgagaacugauaa....................  13    1  seq
..................................................................ucacuggGcggagaacugauaa....................  1     1  seq
..................................................................ucacuggacggagaacugauGa....................  3     1  seq
..................................................................Ncacuggacggagaacugauaa....................  1     1  seq
..................................................................ucacuggacggagaacugauaU....................  8     1  seq
..................................................................ucacuggaAggagaacugauaa....................  1     1  seq
..................................................................ucacuggacggagCacugauaa....................  1     1  seq
..................................................................Acacuggacggagaacugauaag...................  2     1  seq
..................................................................ucacuggacggagaacugauaaU...................  661   1  seq
..................................................................ucacuAgacggagaacugauaag...................  2     1  seq
..................................................................ucacuggacggagaacugauaaC...................  5     1  seq
..................................................................ucacuggacggagaacugauaaA...................  393   1  seq
..................................................................ucacugAacggagaacugauaag...................  1     1  seq
..................................................................ucacAggacggagaacugauaag...................  1     1  seq
..................................................................ucacuggacAgagaacugauaag...................  2     1  seq
```

Star Mature

```
gcguucgcugcaucacucgaucgcacgauuccuuaccauuucuuuguccgguccgugugauuaucaucacuggacggagaacugauaagggccugugacgaccacgag
..................................................................ucacuggacggagaacugauaag...................	511	0	seq
..................................................................uUacuggacggagaacugauaag...................	2	1	seq
..................................................................ucacuggacggagaacugauaagA..................	55	1	seq
..................................................................ucacuggacggagaacugauaagg..................	3	0	seq
..................................................................ucacuggacggagaacugauaagU..................	64	1	seq
..................................................................ucacuggacggagaacugauaaggg.................	3	0	seq
..................................................................ucacuggacggagaacugauaagggc................	2	0	seq
...................................................................cacuggacggagaacugauaa....................	2	0	seq
...................................................................cacuggacggagaaAugauaa....................	1	1	seq
....................................................................Gcuggacggagaacugau......................	1	1	seq
....................................................................acuggacggagaacugauaa....................	4	0	seq
....................................................................acuggacggagaacugauaaU...................	2	1	seq
....................................................................aAuggacggagaacugauaag...................	2	1	seq
....................................................................Ccuggacggagaacugauaagg..................	1	1	seq
....................................................................Ucuggacggagaacugauaagg..................	6	1	seq
....................................................................aAuggacggagaacugauaaggg.................	2	1	seq
....................................................................Ucuggacggagaacugauaaggg.................	2	1	seq
....................................................................aAuggacggagaacugauaagggc................	4	1	seq
....................................................................Ucuggacggagaacugauaagggc................	7	1	seq
.....................................................................cuggacggagaacugauaa....................	2	0	seq
.....................................................................cuggacggagaacugauaag...................	2	0	seq
.....................................................................cuggacggagaacAgauaagg..................	1	1	seq
.....................................................................cAggacggagaacugauaagg..................	1	1	seq
.....................................................................Guggacggagaacugauaagg..................	3	1	seq
.....................................................................cuggacggagaacugauaagA..................	1	1	seq
.....................................................................cuggacggagaacugauaagg..................	140	0	seq
.....................................................................Uuggacggagaacugauaagg..................	3	1	seq
.....................................................................cuggacggagaacugauaaggg.................	51	0	seq
.....................................................................Auggacggagaacugauaaggg.................	3	1	seq
.....................................................................Guggacggagaacugauaaggg.................	4	1	seq
.....................................................................cuggacggagaacugGuaagggc................	1	1	seq
.....................................................................Guggacggagaacugauaagggc................	11	1	seq
.....................................................................Uuggacggagaacugauaagggc................	2	1	seq
.....................................................................cuggacUgagaacugauaagggc................	1	1	seq
.....................................................................Auggacggagaacugauaagggc................	4	1	seq
.....................................................................cuAgacggagaacugauaagggc................	1	1	seq
.....................................................................cuggacggagaacugauaagggc................	404	0	seq
.....................................................................cAggacggagaacugauaagggc................	1	1	seq
.....................................................................cuggacggagaacugauaagggcA...............	2	1	seq
.....................................................................cuggacggagaacugauaagggcU...............	93	1	seq
.....................................................................cuggacggagaacugauaagggcUu..............	3	1	seq
......................................................................uggacggUgaacugauaa....................	5	1	seq
......................................................................uggacgUagaacugauaa....................	1	1	seq
......................................................................uggacggagaacuAauaa....................	24	1	seq
......................................................................uggacggagaaGugauaa....................	1	1	seq
......................................................................uggacgCagaacugauaa....................	1	1	seq
......................................................................uggacggagaacugauUa....................	4	1	seq
......................................................................uggacUgagaacugauaa....................	3	1	seq
......................................................................uggacggagaacugaGaa....................	2	1	seq
......................................................................uggacggaAaacugauaa....................	2	1	seq
......................................................................uggGcggagaacugauaa....................	2	1	seq
......................................................................uggacggaCaacugauaa....................	4	1	seq
......................................................................uCgacggagaacugauaa....................	6	1	seq
......................................................................uggacggagaacGgauaa....................	1	1	seq
......................................................................uggaUggagaacugauaa....................	4	1	seq
......................................................................uggacggagaacugaCaa....................	5	1	seq
......................................................................uggacggagaacugauaC....................	4	1	seq
......................................................................uggacggagaacCgauaa....................	7	1	seq
......................................................................Nggacggagaacugauaa....................	12	1	seq
......................................................................Gggacggagaacugauaa....................	21	1	seq
......................................................................uggacggagaGcugauaa....................	2	1	seq
......................................................................uggacggagaaAugauaa....................	8	1	seq
......................................................................uggacggagGacugauaa....................	4	1	seq
......................................................................uggCcggagaacugauaa....................	2	1	seq
......................................................................uggUcggagaacugauaa....................	2	1	seq
......................................................................uggacggagaacugauaa....................	28107	0	seq
......................................................................ugCacggagaacugauaa....................	5	1	seq
......................................................................uggaAggagaacugauaa....................	2	1	seq
......................................................................uggacgAagaacugauaa....................	6	1	seq
......................................................................uggacggagaacAgauaa....................	12	1	seq
```

```
                              Star                             Mature
gcguucgcugcaucacucgaucgcacgauuccuuaccauuucuuuguccgguccgugugauuaucaucacuggacggagaacugauaagggccugugacgaccacgag
......................................................................uggacggagaacugauGa....................   2       1   seq
......................................................................uggacggagaUcugauaa....................   1       1   seq
......................................................................uAgacggagaacugauaa....................   56      1   seq
......................................................................uggacCgagaacugauaa....................   2       1   seq
......................................................................ugAacggagaacugauaa....................   5       1   seq
......................................................................uggacNgagaacugauaa....................   1       1   seq
......................................................................uUgacggagaacugauaa....................   3       1   seq
......................................................................uggacggagaacugauaU....................   77      1   seq
......................................................................uggacggGgaacugauaa....................   6       1   seq
......................................................................uggacggaUaacugauaa....................   1       1   seq
......................................................................uggacggagaacuUauaa....................   3       1   seq
......................................................................uggacggagaacuCauaa....................   3       1   seq
......................................................................uggacggagaacugUuaa....................   2       1   seq
......................................................................uggacggagUacugauaa....................   9       1   seq
......................................................................uggacggagaacugGuaa....................   1       1   seq
......................................................................Aggacggagaacugauaa....................   118     1   seq
......................................................................uggacggagaaUugauaa....................   1       1   seq
......................................................................ugUacggagaacugauaa....................   1       1   seq
......................................................................uggacAgagaacugauaa....................   44      1   seq
......................................................................uggacggagaacugaAaa....................   66      1   seq
......................................................................Cggacggagaacugauaa....................   1       1   seq
......................................................................uggacggagaGcugauaag...................   25      1   seq
......................................................................uggacggagaacGgauaag...................   3       1   seq
......................................................................uggacAgagaacugauaag...................   165     1   seq
......................................................................uggacggagaacugauaag...................   103608  0   seq
......................................................................uUgacggagaacugauaag...................   11      1   seq
......................................................................uggaAggagaacugauaag...................   12      1   seq
......................................................................uggacggaCaacugauaag...................   4       1   seq
......................................................................uggacggagaacuCauaag...................   7       1   seq
......................................................................uggacggagaacugauCag...................   3       1   seq
......................................................................uggGcggagaacugauaag...................   17      1   seq
......................................................................uggacggagaacugauaUg...................   28      1   seq
......................................................................uggacggagaacuAauaag...................   72      1   seq
......................................................................uggacggagaacugauaaA...................   13769   1   seq
......................................................................uggacggagaacuUauaag...................   9       1   seq
......................................................................uggacggaUaacugauaag...................   6       1   seq
......................................................................uggacggagaacugauUag...................   7       1   seq
......................................................................uggacggagaacugaCaag...................   10      1   seq
......................................................................uggacggagaacugauGag...................   33      1   seq
......................................................................uggacggagaUcugauaag...................   15      1   seq
......................................................................Nggacggagaacugauaag...................   33      1   seq
......................................................................uggCcggagaacugauaag...................   6       1   seq
......................................................................Cggacggagaacugauaag...................   10      1   seq
......................................................................uggacCgagaacugauaag...................   14      1   seq
......................................................................uggacggagaacCgauaag...................   7       1   seq
......................................................................ugUacggagaacugauaag...................   12      1   seq
......................................................................uggacgUagaacugauaag...................   17      1   seq
......................................................................uAgacggagaacugauaag...................   225     1   seq
......................................................................uggacUgagaacugauaag...................   17      1   seq
......................................................................uggacggagaaGugauaag...................   6       1   seq
......................................................................uggacggagaacugauaaU...................   2967    1   seq
......................................................................uggacggagaacugUuaag...................   22      1   seq
......................................................................uggacggagaaUugauaag...................   35      1   seq
......................................................................uggacggaAaacugauaag...................   20      1   seq
......................................................................uggacggUgaacugauaag...................   13      1   seq
......................................................................uggacggagaacugaGaag...................   7       1   seq
......................................................................ugCacggagaacugauaag...................   26      1   seq
......................................................................Gggacggagaacugauaag...................   72      1   seq
......................................................................uggacggagaacugauaaC...................   88      1   seq
......................................................................ugAacggagaacugauaag...................   11      1   seq
......................................................................uggacggagaacAgauaag...................   26      1   seq
......................................................................uggacggGgaacugauaag...................   28      1   seq
......................................................................uggacNgagaacugauaag...................   3       1   seq
......................................................................uggacgAagaacugauaag...................   9       1   seq
......................................................................uggacggagaaAugauaag...................   19      1   seq
......................................................................uCgacggagaacugauaag...................   21      1   seq
......................................................................uggacggagUacugauaag...................   12      1   seq
......................................................................uggaGggagaacugauaag...................   4       1   seq
......................................................................uggacggagCacugauaag...................   1       1   seq
......................................................................uggacggagaacugGuaag...................   14      1   seq
```

Star Mature

```
gcguucgcugcaucacucgaucgcacgauuccuuaccauuucuuuguccgguccgugugauuaucaucacuggacggagaacugauaagggccugugacgaccacgag
......................................................................uNgacggagaacugauaag...................   4        1    seq
......................................................................uggacgCagaacugauaag...................   11       1    seq
......................................................................uggaUggagaacugauaag...................   32       1    seq
......................................................................uggUcggagaacugauaag...................   6        1    seq
......................................................................Aggacggagaacugauaag...................   387      1    seq
......................................................................uggacggagaacugaAaag...................   76       1    seq
......................................................................uggacggagGacugauaag...................   14       1    seq
......................................................................uggacggagaacGgauaagg..................   39       1    seq
......................................................................uggacggagaacAgauaagg..................   196      1    seq
......................................................................uggacggGgaacugauaagg..................   154      1    seq
......................................................................uggacggagaacugauCagg..................   4        1    seq
......................................................................uggacNgagaacugauaagg..................   18       1    seq
......................................................................uggacAgagaacugauaagg..................   1116     1    seq
......................................................................Nggacggagaacugauaagg..................   214      1    seq
......................................................................uUgacggagaacugauaagg..................   70       1    seq
......................................................................uggacggagUacugauaagg..................   96       1    seq
......................................................................uggacggagaacugaGaagg..................   41       1    seq
......................................................................ugNacggagaacugauaagg..................   2        1    seq
......................................................................uggacggaCaacugauaagg..................   38       1    seq
......................................................................Cggacggagaacugauaagg..................   92       1    seq
......................................................................uggacggagaacuAauaagg..................   414      1    seq
......................................................................uggacggaUaacugauaagg..................   39       1    seq
......................................................................uggacggagaCcugauaagg..................   9        1    seq
......................................................................uggacgCagaacugauaagg..................   42       1    seq
......................................................................uggacggagaacugauNagg..................   1        1    seq
......................................................................uggacggagaaGugauaagg..................   15       1    seq
......................................................................uggGcggagaacugauaagg..................   160      1    seq
......................................................................uggacggCgaacugauaagg..................   11       1    seq
......................................................................Gggacggagaacugauaagg..................   446      1    seq
......................................................................uggacggagaacugauaagC..................   153      1    seq
......................................................................uggacggagNacugauaagg..................   3        1    seq
......................................................................uggacggagaacugauaaAg..................   417      1    seq
......................................................................uggacggagaacCgauaagg..................   120      1    seq
......................................................................uggacggagaacugauaaUg..................   36       1    seq
......................................................................uggacggagaUcugauaagg..................   97       1    seq
......................................................................ugAacggagaacugauaagg..................   135      1    seq
......................................................................ugUacggagaacugauaagg..................   73       1    seq
......................................................................uggacggagaNcugauaagg..................   1        1    seq
......................................................................uggacggagaacugauaagA..................   33637    1    seq
......................................................................uggacUgagaacugauaagg..................   123      1    seq
......................................................................uggacggagaaAugauaagg..................   155      1    seq
......................................................................uAgacggagaacugauaagg..................   1532     1    seq
......................................................................uggacggagaacuUauaagg..................   64       1    seq
......................................................................uggacggagaacugauGagg..................   203      1    seq
......................................................................uggacggagaacugaCaagg..................   69       1    seq
......................................................................uggacggagaacugaAaagg..................   417      1    seq
......................................................................uggacggagaacugUuaagg..................   84       1    seq
......................................................................uggacggagGacugauaagg..................   109      1    seq
......................................................................ugCacggagaacugauaagg..................   190      1    seq
......................................................................uggacggagaacugauaaCg..................   30       1    seq
......................................................................uggacggagaacugauaagg..................   749669   0    seq
......................................................................uggacggaAaacugauaagg..................   56       1    seq
......................................................................uggacggagaacugGuaagg..................   74       1    seq
......................................................................uggacggagaGcugauaagg..................   376      1    seq
......................................................................uggaGggagaacugauaagg..................   27       1    seq
......................................................................uggacggUgaacugauaagg..................   107      1    seq
......................................................................Aggacggagaacugauaagg..................   2851     1    seq
......................................................................uggacggagaacugauaagU..................   2280     1    seq
......................................................................uggaUggagaacugauaagg..................   146      1    seq
......................................................................uggacggagaacugauUagg..................   20       1    seq
......................................................................uggCcggagaacugauaagg..................   38       1    seq
......................................................................uCgacggagaacugauaagg..................   107      1    seq
......................................................................uggacggagaacuNauaagg..................   2        1    seq
......................................................................uggacggagCacugauaagg..................   14       1    seq
......................................................................uggacCgagaacugauaagg..................   69       1    seq
......................................................................uggaAggagaacugauaagg..................   101      1    seq
......................................................................uggacggagaacugCuaagg..................   5        1    seq
......................................................................uggacgUagaacugauaagg..................   86       1    seq
......................................................................uggUcggagaacugauaagg..................   102      1    seq
......................................................................uNgacggagaacugauaagg..................   23       1    seq
```

```
                              Star                                    Mature
gcguucgcugcaucacucgaucgcacgauuccuuaccauuucuuuguccgguccgugugauuaucaucacuggacggagaacugauaagggccugugacgaccacgag
......................................................................uggacggagaacuCauaagg..................	43	1	seq
......................................................................uggacggagaaUugauaagg..................	40	1	seq
......................................................................uggacgNagaacugauaagg..................	1	1	seq
......................................................................uggacggagaacugauaUgg..................	66	1	seq
......................................................................uggacggagaacugauaCgg..................	21	1	seq
......................................................................uggacgAagaacugauaagg..................	126	1	seq
......................................................................uggacggaNaacugauaagg..................	2	1	seq
......................................................................uggacggagaacGgauaaggg.................	33	1	seq
......................................................................uggacggagaacuCauaaggg.................	43	1	seq
......................................................................uggacggagaacugauaaggN.................	3	1	seq
......................................................................uggaUggagaacugauaaggg.................	128	1	seq
......................................................................uggacggagaacugaGaaggg.................	61	1	seq
......................................................................uggacggagaacugauaUggg.................	50	1	seq
......................................................................uggGcggagaacugauaaggg.................	143	1	seq
......................................................................uUgacggagaacugauaaggg.................	74	1	seq
......................................................................uggacggagaacugauUaggg.................	28	1	seq
......................................................................uggacggaCaacugauaaggg.................	27	1	seq
......................................................................uggacggagaUcugauaaggg.................	89	1	seq
......................................................................uAgacggagaacugauaaggg.................	1654	1	seq
......................................................................uggacggagaacugauGaggg.................	163	1	seq
......................................................................uCgacggagaacugauaaggg.................	116	1	seq
......................................................................Cggacggagaacugauaaggg.................	175	1	seq
......................................................................uggacggagaacAgauaaggg.................	200	1	seq
......................................................................uggacggagaacugCuaaggg.................	5	1	seq
......................................................................uggacggagaacugauaagUg.................	90	1	seq
......................................................................uggacggagaGcugauaaggg.................	459	1	seq
......................................................................uggacggagaacugaCaaggg.................	94	1	seq
......................................................................Gggacggagaacugauaaggg.................	495	1	seq
......................................................................uggaGggagaacugauaaggg.................	24	1	seq
......................................................................uggacNgagaacugauaaggg.................	22	1	seq
......................................................................uNgacggagaacugauaaggg.................	18	1	seq
......................................................................ugCacggagaacugauaaggg.................	191	1	seq
......................................................................uggacggagaacugauaaAgg.................	814	1	seq
......................................................................uggacggagaacuNauaaggg.................	1	1	seq
......................................................................uggacggCgaacugauaaggg.................	2	1	seq
......................................................................uggacCgagaacugauaaggg.................	50	1	seq
......................................................................uggacggUgaacugauaaggg.................	122	1	seq
......................................................................uggacggagaaAugauaaggg.................	132	1	seq
......................................................................ugAacggagaacugauaaggg.................	131	1	seq
......................................................................uggacggagaacugaAaaggg.................	315	1	seq
......................................................................uggacggagCacugauaaggg.................	10	1	seq
......................................................................uggaAggagaacugauaaggg.................	90	1	seq
......................................................................uggacggagaCcugauaaggg.................	7	1	seq
......................................................................uggacggagNacugauaaggg.................	4	1	seq
......................................................................Aggacggagaacugauaaggg.................	2685	1	seq
......................................................................uggacggagaacugauaagCg.................	38	1	seq
......................................................................uggacggagaacCgauaaggg.................	128	1	seq
......................................................................uggacggagGacugauaaggg.................	117	1	seq
......................................................................uggacAgagaacugauaaggg.................	1188	1	seq
......................................................................uggacggagaacugauaaggg.................	726957	0	seq
......................................................................uggacUgagaacugauaaggg.................	107	1	seq
......................................................................uggacggGgaacugauaaggg.................	132	1	seq
......................................................................uggacggagaacugUuaaggg.................	88	1	seq
......................................................................uggacgCagaacugauaaggg.................	55	1	seq
......................................................................uggUcggagaacugauaaggg.................	84	1	seq
......................................................................uggacggagaacugauaCggg.................	4	1	seq
......................................................................uggacggagaacugauCaggg.................	2	1	seq
......................................................................uggacggagUacugauaaggg.................	94	1	seq
......................................................................uggacgAagaacugauaaggg.................	124	1	seq
......................................................................uggacggagaacugauaaggA.................	93833	1	seq
......................................................................uggacggagaacugaNaaggg.................	1	1	seq
......................................................................uggacggagaacugauaaggC.................	966	1	seq
......................................................................uggacggagaaUugauaaggg.................	47	1	seq
......................................................................uggacggagaacugauaagAg.................	617	1	seq
......................................................................uggacggagaaGugauaaggg.................	24	1	seq
......................................................................Nggacggagaacugauaaggg.................	208	1	seq
......................................................................uggacggaUaacugauaaggg.................	106	1	seq
......................................................................uggacggagaacuAauaaggg.................	492	1	seq
......................................................................ugUacggagaacugauaaggg.................	71	1	seq
......................................................................uggacggagaacugauaaggU.................	7150	1	seq
```

```
                                                      Star                                          Mature
gcguucgcugcaucacucgaucgcacgauuccuuaccauuucuuuguccgguccgugugauuaucaucacuggacggagaacugauaagggccugugacgaccacgag
......................................................................uggacggaAacugauaaggg.................	66	1	seq
......................................................................uggacggagaacugauaaCgg.................	24	1	seq
......................................................................uggacgUagaacugauaaggg.................	87	1	seq
......................................................................uggacggagaacugauNaggg.................	2	1	seq
......................................................................uggacggagaacugauaaUgg.................	50	1	seq
......................................................................uggacggagaacugauaGggg.................	266	1	seq
......................................................................uggacggagaacugGuaaggg.................	80	1	seq
......................................................................uggCcggagaacugauaaggg.................	33	1	seq
......................................................................uggacggagaacuUauaaggg.................	74	1	seq
......................................................................uggacggagaacCgauaagggc................	779	1	seq
......................................................................uggacggagaacGgauaagggc................	236	1	seq
......................................................................uggacAgagaacugauaagggc................	8379	1	seq
......................................................................uggacggagaUcugauaagggc................	574	1	seq
......................................................................ugAacggagaacugauaagggc................	889	1	seq
......................................................................uggCcggagaacugauaagggc................	227	1	seq
......................................................................Cggacggagaacugauaagggc................	689	1	seq
......................................................................uggacggagaacugauaagggc................	4493635	0	seq
......................................................................uggacggagaacugauaagggA................	128972	1	seq
......................................................................uggacggagaacugauaagUgc................	382	1	seq
......................................................................ugCacggagaacugauaagggc................	1162	1	seq
......................................................................uggacggagaacugauaaUggc................	294	1	seq
......................................................................uggacggagaacugauaaggUc................	451	1	seq
......................................................................uggacgCagaacugauaagggc................	316	1	seq
......................................................................uggacggagaacugaCaagggc................	522	1	seq
......................................................................uggacggagaNcugauaagggc................	6	1	seq
......................................................................uggacggagaacugauaaCggc................	178	1	seq
......................................................................uggaUggagaacugauaagggc................	892	1	seq
......................................................................uggacggagaacugauaGgggc................	1194	1	seq
......................................................................uggacggagCacugauaagggc................	65	1	seq
......................................................................uggaGggagaacugauaagggc................	268	1	seq
......................................................................uggGcggagaacugauaagggc................	835	1	seq
......................................................................uggacggagaacugauNagggc................	6	1	seq
......................................................................uggacggagaacugCuaagggc................	22	1	seq
......................................................................uggacggaAacugauaagggc................	504	1	seq
......................................................................uggacggagaacugauaNgggc................	1	1	seq
......................................................................uggacggagaacugGuaagggc................	546	1	seq
......................................................................uUgacggagaacugauaagggc................	419	1	seq
......................................................................uggacggagaaUugauaagggc................	485	1	seq
......................................................................uggacggagaacugaAaagggc................	2093	1	seq
......................................................................uggaNggagaacugauaagggc................	1	1	seq
......................................................................uggacggagaacugaNaagggc................	2	1	seq
......................................................................uggacggaCaacugauaagggc................	213	1	seq
......................................................................uggacggCgaacugauaagggc................	49	1	seq
......................................................................uggacggGgaacugauaagggc................	894	1	seq
......................................................................uggacggagaacuAauaagggc................	3915	1	seq
......................................................................uggacggagaacAgauaagggc................	1379	1	seq
......................................................................uggacggagaacugauaaAggc................	3391	1	seq
......................................................................uggacggagGacugauaagggc................	721	1	seq
......................................................................Nggacggagaacugauaagggc................	1228	1	seq
......................................................................ugNacggagaacugauaagggc................	10	1	seq
......................................................................uNgacggagaacugauaagggc................	114	1	seq
......................................................................uggacggagaacugauaaggAc................	875	1	seq
......................................................................uggacggagaaAugauaagggc................	810	1	seq
......................................................................uggacggagaaGugauaagggc................	150	1	seq
......................................................................uggacggagaacuNauaagggc................	6	1	seq
......................................................................uggacggaNaacugauaagggc................	5	1	seq
......................................................................uggacggagaacugUuaagggc................	590	1	seq
......................................................................uggacNgagaacugauaagggc................	101	1	seq
......................................................................uggacggagaacugauaaggCc................	1104	1	seq
......................................................................uggacggagaacugauaagNgc................	5	1	seq
......................................................................Gggacggagaacugauaagggc................	3478	1	seq
......................................................................uCgacggagaacugauaagggc................	741	1	seq
......................................................................uggacggagaacugauaagggN................	8	1	seq
......................................................................uggacggagUacugauaagggc................	538	1	seq
......................................................................Aggacggagaacugauaagggc................	14504	1	seq
......................................................................uggaAggagaacugauaagggc................	617	1	seq
......................................................................uggacggagaacugauaaggNc................	22	1	seq
......................................................................uggacggUgaacugauaagggc................	749	1	seq
......................................................................uggacggagaCcugauaagggc................	31	1	seq
......................................................................uggacgUagaacugauaagggc................	479	1	seq
```

Star Mature

```
gcguucgcugcaucacucgaucgcacgauuccuuaccauuucuuuguccgguccgugugauuaucaucacuggacggagaacugauaagggccugugacgaccacgag
......................................................................uggacggagaGcugauaagggc................ 928 1 seq
......................................................................uggacggagaacugauaCgggc................ 39 1 seq
......................................................................uggacggagaacugauaagCgc................ 283 1 seq
......................................................................uggacggagNacugauaagggc................ 16 1 seq
......................................................................uggacgNagaacugauaagggc................ 1 1 seq
......................................................................uggacggagaacugauaagAgc................ 2307 1 seq
......................................................................uggacggagaacugauCagggc................ 12 1 seq
......................................................................uggacgAagaacugauaagggc................ 799 1 seq
......................................................................uggacggagaacugaGaagggc................ 231 1 seq
......................................................................uggacggagaacugauaaNggc................ 3 1 seq
......................................................................uggacggagaacugauaagggU................ 42431 1 seq
......................................................................uggacggagaacugauaUgggc................ 408 1 seq
......................................................................uggacggagaacugauUagggc................ 170 1 seq
......................................................................uggacggaUaacugauaagggc................ 198 1 seq
......................................................................uAgacggagaacugauaagggc................ 11622 1 seq
......................................................................uggacUgagaacugauaagggc................ 677 1 seq
......................................................................uggacggagaacugauGagggc................ 1349 1 seq
......................................................................uggacggagaacugauaagggG................ 1037 1 seq
......................................................................uggacggagaaNugauaagggc................ 1 1 seq
......................................................................ugUacggagaacugauaagggc................ 400 1 seq
......................................................................uggUcggagaacugauaagggc................ 560 1 seq
......................................................................uggacggagaacuCauaagggc................ 244 1 seq
......................................................................uggacCgagaacugauaagggc................ 416 1 seq
......................................................................uggacggagaacuUauaagggc................ 408 1 seq
......................................................................uggacggagaacugauaagggUc............... 65 1 seq
......................................................................uggacggagaacugauaagggcN............... 6 1 seq
......................................................................uggacAgagaacugauaagggcc............... 1 1 seq
......................................................................uggacggagaacugaAaagggcc............... 1 1 seq
......................................................................uggacggagaacugauaagggcG............... 1048 1 seq
......................................................................uggacggagaacugauaagggAc............... 16 1 seq
......................................................................uggaUggagaacugauaagggcc............... 1 1 seq
......................................................................uggacggagaacugauaagggcA............... 140331 1 seq
......................................................................uggacggagaacugauaagggGc............... 1 1 seq
......................................................................uggacggagaacugauaagggcc............... 319 0 seq
......................................................................uggacggagaacugauaagggcU............... 133677 1 seq
......................................................................uggacggagaacugauaagggcAu.............. 20679 1 seq
......................................................................uggacggagaacugauaagggccG.............. 2 1 seq
......................................................................uggacggagaacugauaagggUcu.............. 1 1 seq
......................................................................uggacggagaacugauaagggccu.............. 39 0 seq
......................................................................uggacggagaacugauaagggcGu.............. 14 1 seq
......................................................................uggacggagaacugauaaUggccu.............. 1 1 seq
......................................................................uggacggagaacugauaagggcUu.............. 17941 1 seq
......................................................................uggacggagaacugauaagggccA.............. 31 1 seq
......................................................................uggacggagaacugauaagggcNu.............. 1 1 seq
......................................................................uggacggagaacugauaagggccuU............. 5 1 seq
......................................................................uggacggagaacugauaagggcAug............. 3 1 seq
......................................................................uggacggagaacugauaagggcUug............. 3 1 seq
......................................................................uggacggagaacugauaagggcUugu............ 13 1 seq
......................................................................uggacggagaacugauaagggccuAu............ 1 1 seq
......................................................................uggacggagaacugauaagggccuUu............ 9 1 seq
.......................................................................ggacggagaacugauaag................... 349 0 seq
.......................................................................ggacggagaaAugauaag................... 1 1 seq
.......................................................................ggacggagaacugaAaag................... 3 1 seq
.......................................................................ggacggagaacugauaaA................... 41 1 seq
.......................................................................ggacggagaacAgauaag................... 2 1 seq
.......................................................................ggacggagaacugauaaU................... 2 1 seq
.......................................................................Ugacggagaacugauaag................... 10 1 seq
.......................................................................ggacggagCacugauaag................... 1 1 seq
.......................................................................ggUcggagaacugauaag................... 1 1 seq
.......................................................................ggGcggagaacugauaag................... 11 1 seq
.......................................................................ggacAgagaacugauaagg.................. 1 1 seq
.......................................................................ggacggagaacugauaagU.................. 2 1 seq
.......................................................................ggacggagaacAgauaagg.................. 26 1 seq
.......................................................................ggacggagaacugauUagg.................. 1 1 seq
.......................................................................Cgacggagaacugauaagg.................. 1 1 seq
.......................................................................ggaUggagaacugauaagg.................. 1 1 seq
.......................................................................Ugacggagaacugauaagg.................. 47 1 seq
.......................................................................ggacCgagaacugauaagg.................. 3 1 seq
.......................................................................ggacggagaacugauaUgg.................. 1 1 seq
.......................................................................ggCcggagaacugauaagg.................. 1 1 seq
```

Star Mature

```
gcguucgcugcaucacucgaucgcacgauuccuuaccauuucuuuguccgguccgugugauuaucaucacuggacggagaacugauaagggccugugacgaccacgag
.......................................................................ggacggagaacGgauaagg..................  1     1  seq
.......................................................................gCacggagaacugauaagg..................  1     1  seq
.......................................................................ggacggagaacugauaaUg..................  4     1  seq
.......................................................................Agacggagaacugauaagg..................  1     1  seq
.......................................................................ggacggagCacugauaagg..................  10    1  seq
.......................................................................ggacggagaacugauaaAg..................  2     1  seq
.......................................................................ggacggagaacuAauaagg..................  1     1  seq
.......................................................................gAacggagaacugauaagg..................  1     1  seq
.......................................................................ggacggagaacugaAaagg..................  2     1  seq
.......................................................................Ngacggagaacugauaagg..................  1     1  seq
.......................................................................ggacggagaaAugauaagg..................  2     1  seq
.......................................................................ggacggagaacugauaagg..................  2265  0  seq
.......................................................................ggGcggagaacugauaagg..................  79    1  seq
.......................................................................ggacggagaacugauGagg..................  1     1  seq
.......................................................................ggacggagGacugauaagg..................  2     1  seq
.......................................................................ggacggaAaacugauaagg..................  1     1  seq
.......................................................................ggacggagaGcugauaagg..................  1     1  seq
.......................................................................ggacggagaacCgauaagg..................  1     1  seq
.......................................................................ggacggagaacugauaagA..................  128   1  seq
.......................................................................ggacggagaacugauaagAg.................  3     1  seq
.......................................................................ggacggagaacCgauaaggg.................  1     1  seq
.......................................................................ggacggagaaAugauaaggg.................  2     1  seq
.......................................................................ggacggagaacugGuaaggg.................  1     1  seq
.......................................................................ggacggagaacugaGaaggg.................  1     1  seq
.......................................................................ggacggagGacugauaaggg.................  4     1  seq
.......................................................................ggacggagaacugauaaggC.................  4     1  seq
.......................................................................ggacggagaacugauaaUgg.................  2     1  seq
.......................................................................ggacggagCacugauaaggg.................  18    1  seq
.......................................................................ggacggagaacugauaGggg.................  1     1  seq
.......................................................................ggaAggagaacugauaaggg.................  2     1  seq
.......................................................................ggacggagaacGgauaaggg.................  1     1  seq
.......................................................................ggacggagaacuAauaaggg.................  2     1  seq
.......................................................................ggacggagaacAgauaaggg.................  28    1  seq
.......................................................................ggacggagaacugauaaAgg.................  1     1  seq
.......................................................................Ugacggagaacugauaaggg.................  32    1  seq
.......................................................................ggacCgagaacugauaaggg.................  3     1  seq
.......................................................................Agacggagaacugauaaggg.................  1     1  seq
.......................................................................ggacggagaacugauaaggA.................  332   1  seq
.......................................................................ggacgCagaacugauaaggg.................  1     1  seq
.......................................................................ggacAgagaacugauaaggg.................  3     1  seq
.......................................................................ggGcggagaacugauaaggg.................  90    1  seq
.......................................................................ggacggagaGcugauaaggg.................  2     1  seq
.......................................................................ggacggagaacugauaaggg.................  2520  0  seq
.......................................................................ggacggagaacugauaaggU.................  59    1  seq
.......................................................................ggacggagaacugaAaaggg.................  5     1  seq
.......................................................................ggacggagaacGgauaagggc................  5     1  seq
.......................................................................ggacggagCacugauaagggc................  56    1  seq
.......................................................................ggacggagUacugauaagggc................  1     1  seq
.......................................................................Ngacggagaacugauaagggc................  5     1  seq
.......................................................................ggacggagaacugauGagggc................  2     1  seq
.......................................................................ggacggagaacugauaagggU................  159   1  seq
.......................................................................ggacggagaacugaAaagggc................  27    1  seq
.......................................................................ggacggagaGcugauaagggc................  1     1  seq
.......................................................................ggacggagaacugCuaagggc................  1     1  seq
.......................................................................ggaAggagaacugauaagggc................  1     1  seq
.......................................................................ggacggUgaacugauaagggc................  1     1  seq
.......................................................................ggacggagGacugauaagggc................  7     1  seq
.......................................................................ggacggagaacugauaagUgc................  1     1  seq
.......................................................................ggacggagaacugaCaagggc................  2     1  seq
.......................................................................ggacggagaacugauaGgggc................  1     1  seq
.......................................................................ggUcggagaacugauaagggc................  1     1  seq
.......................................................................ggacggagaacugauaaggAc................  1     1  seq
.......................................................................ggacggagaacugauaagAgc................  6     1  seq
.......................................................................ggacggagaacugUuaagggc................  7     1  seq
.......................................................................ggacggagaacuAauaagggc................  8     1  seq
.......................................................................ggacggagaacugauUagggc................  4     1  seq
.......................................................................ggacggagaaAugauaagggc................  35    1  seq
.......................................................................ggacggagaacuUauaagggc................  3     1  seq
.......................................................................Agacggagaacugauaagggc................  2     1  seq
.......................................................................ggacggagaacugGuaagggc................  3     1  seq
```

```
                              Star                            Mature
gcguucgcugcaucacucgaucgcacgauuccuuaccauuucuuuguccgguccgugugauuaucaucacuggacggagaacugauaagggccugugacgaccacgag
.......................................................................ggacAgagaacugauaagggc................   14      1    seq
.......................................................................ggacggagaacugauaUgggc................   1       1    seq
.......................................................................ggacggaAaacugauaagggc................   3       1    seq
.......................................................................ggacggagaacugauaaUggc................   16      1    seq
.......................................................................ggacgAagaacugauaagggc................   1       1    seq
.......................................................................ggacggGgaacugauaagggc................   2       1    seq
.......................................................................gUacggagaacugauaagggc................   2       1    seq
.......................................................................ggacggagaacCgauaagggc................   1       1    seq
.......................................................................ggacggagaacAgauaagggc................   172     1    seq
.......................................................................Ugacggagaacugauaagggc................   328     1    seq
.......................................................................ggacggCgaacugauaagggc................   2       1    seq
.......................................................................ggacggagaacugauaaCggc................   2       1    seq
.......................................................................ggacggagaacugauaagggA................   327     1    seq
.......................................................................ggGcggagaacugauaagggc................   560     1    seq
.......................................................................ggacggagaaUugauaagggc................   1       1    seq
.......................................................................ggacggagaacugauaaggUc................   1       1    seq
.......................................................................gAacggagaacugauaagggc................   4       1    seq
.......................................................................ggacCgagaacugauaagggc................   9       1    seq
.......................................................................ggacgUagaacugauaagggc................   1       1    seq
.......................................................................ggacggaCaacugauaagggc................   1       1    seq
.......................................................................ggacggagaacugauaaggCc................   2       1    seq
.......................................................................ggacggagaacugauaagggG................   3       1    seq
.......................................................................ggacggagaacugauaaAggc................   10      1    seq
.......................................................................ggacggagaacugauaagggc................   13122   0    seq
.......................................................................ggacggagaacugauaagggcU...............   465     1    seq
.......................................................................ggacggagaacugauaagggcG...............   20      1    seq
.......................................................................ggacggagaacugauaagggcA...............   392     1    seq
.......................................................................ggacggagaacugauaagggcAu..............   110     1    seq
.......................................................................ggacggagaacugauaagggcUu..............   10      1    seq
.......................................................................ggacggagaacugauaagggcUugu............   1       1    seq
........................................................................Uacggagaacugauaagg..................   1       1    seq
........................................................................Uacggagaacugauaaggg.................   4       1    seq
........................................................................gacggagaacugauaaggg.................   1       0    seq
........................................................................Uacggagaacugauaagggc................   5       1    seq
........................................................................gGcggagaacugauaagggc................   6       1    seq
........................................................................gacggaNaacugauaagggc................   1       1    seq
........................................................................gacggagaacugauaagggc................   582     0    seq
........................................................................gacggagaacugauaagggU................   1       1    seq
........................................................................gacggagaacugauaagggA................   1       1    seq
........................................................................gacggagaacugauaagggcA...............   2       1    seq
........................................................................gacggagaacugauaagggcU...............   55      1    seq
........................................................................gacggagaacugauaagggcUu..............   8       1    seq
.........................................................................acggagaacugauaaggg.................   2       0    seq
.........................................................................Ucggagaacugauaagggc................   1       1    seq
.........................................................................acggagaacugauaagggc................   140     0    seq
.........................................................................acggagaacugauaagggcA...............   3       1    seq
.........................................................................acggagaacugauaagggcUu..............   1       1    seq
..........................................................................cggagaacugauaagggc................   107     0    seq
..........................................................................Aggagaacugauaagggc................   4       1    seq
..........................................................................Uggagaacugauaagggc................   27      1    seq
..........................................................................cAgagaacugauaagggc................   1       1    seq
..........................................................................cggagaacugauaagggcA...............   4       1    seq
..........................................................................cggagaacugauaagggcU...............   2       1    seq
..........................................................................cggagaacugauaagggcAu..............   2       1    seq
...........................................................................ggagaacugauaagggcA...............   3       1    seq
............................................................................gagaacugauaagggcAu..............   2       1    seq
```
